# Congenital Transmission of *Trypanosoma cruzi*: A Review About the Interactions Between the Parasite, the Placenta, the Maternal and the Fetal/Neonatal Immune Responses

**DOI:** 10.3389/fmicb.2019.01854

**Published:** 2019-08-14

**Authors:** Ulrike Kemmerling, Antonio Osuna, Alejandro Gabriel Schijman, Carine Truyens

**Affiliations:** ^1^Programa de Anatomía y Biología del Desarrollo, Instituto de Ciencias Biomédicas, Facultad de Medicina, Universidad de Chile, Santiago, Chile; ^2^Grupo de Bioquímica y Parasitología Molecular, Departamento de Parasitología, Instituto de Biotecnología, Universidad de Granada, Granada, Spain; ^3^Molecular Biology of Chagas Disease Laboratory, Genetic Engineering and Molecular Biology Research Institute Dr. Héctor Torres (INGEBI-CONICET), Buenos Aires, Argentina; ^4^Laboratory of Parasitology, Faculty of Medicine, Université Libre de Bruxelles, Brussels, Belgium

**Keywords:** *Trypanosoma cruzi*, infection, maternal-fetal interactions, placenta, congenital chagas disease

## Abstract

Chagas disease (CD), caused by the protozoan parasite *Trypanosoma cruzi*, is considered a neglected tropical disease by the World Health Organization. Congenital transmission of CD is an increasingly relevant public health problem. It progressively becomes the main transmission route over others and can occur in both endemic and non-endemic countries. Though most congenitally infected newborns are asymptomatic at birth, they display higher frequencies of prematurity, low birth weight, and lower Apgar scores compared to uninfected ones, and some suffer from severe symptoms. If not diagnosed and treated, infected newborns are at risk of developing disabling and life-threatening chronic pathologies later in life. The success or failure of congenital transmission depends on interactions between the parasite, the placenta, the mother, and the fetus. We review and discuss here the current knowledge about these parameters, including parasite virulence factors such as exovesicles, placental tropism, potential placental defense mechanisms, the placental transcriptome of infected women, gene polymorphism, and the maternal and fetal/neonatal immune responses, that might modulate the risk of *T. cruzi* congenital transmission.

## Chagas Disease

*Trypanosoma cruzi*, a protozoan parasite, is the etiological agent of CD, a neglected tropical disease (WHO, 2015; Lenk et al., 2018) endemic to Latin American countries. Affected countries extend from the United States to Argentina and Chile (Pérez-Molina and Molina, 2018). The parasite is primarily transmitted by insect vectors, but can also be transmitted through blood transfusion, organ transplantation, consumption of parasite-contaminated food, and vertically from mother to child. In Latin America, CD is an important cause of cardiovascular morbidity and premature death (Rassi et al., 2012; WHO, 2015).

The disease evolves in two phases. The acute phase, defined by high parasitemia, lasts 2–3 months. It is often asymptomatic or involves non-specific flu-like symptoms. However, 2–12% of the infected individuals, mostly children under 3 years, die from acute myocardiopathy at times associated with a meningoencephalic compromise (Carlier and Truyens, 2015). The immune response eliminates most but not all parasites. As a consequence, the individual enters the chronic phase and remains infected for the rest of their lives. Although most patients remain asymptomatic for periods ranging in length from several months to decades, 30 – 40% of the infected people develop cardiac (mostly) and/or digestive tract (less frequent) pathologies that may lead to premature death (Rassi et al., 2012; de Oliveira et al., 2018).

## Congenital Chagas Disease

Most women of gestational age are chronically infected, having acquired the infection during infancy. Around 5% of them transmit the parasite to their fetus, and thus approximately 9000 newborns infected with CD in Latin America are born every year. Cases of congenital CD have also been reported outside of the naturally endemic area (WHO, 2015; Antinori et al., 2017). Congenital infection is gaining importance as a route of *T. cruzi* transmission in endemic countries, due to control programs of vectorial and transfusional transmission routes, and is now estimated to account for 22% of new cases (WHO, 2015; Picado et al., 2018). In non-endemic countries, most of the new cases result from congenital transmission (Howard et al., 2014; WHO, 2015). Importantly, since *T. cruzi* maternal-fetal transmission can be repeated at each pregnancy and observed from one generation to another, this way of transmission can easily expand in time (Messenger et al., 2017).

Congenital CD is characteristically an acute parasite infection. Though most (around 60%) congenitally infected newborns are asymptomatic at birth, they display higher frequencies of low birth weight, prematurity, and lower Apgar scores at birth compared to uninfected newborns, while some infected newborns suffer from severe symptoms that can rapidly lead to death (Torrico et al., 2004; Liempi et al., 2016). Moreover, congenitally infected infants are at risk of disabling and life-threatening chronic pathologies later in life (Cardoso et al., 2012; Requena-Méndez et al., 2014; Carlier et al., 2015; Antinori et al., 2017). It is therefore essential to prevent congenital transmission and to rapidly diagnose and treat congenitally infected newborns.

Importantly, the occurrence of congenital transmission depends on interactions between *T. cruzi*, the placenta, the maternal immune system, and the developing fetal/neonatal immune response (Carlier and Truyens, 2015; Liempi et al., 2016). Here we will discuss the factors mentioned above that modulate the risk and probability of *T. cruzi* maternal-fetal transmission.

## The Parasite Diversity

*Trypanosoma cruzi* belongs to the Kinetoplastida order, Trypanosomatidae family and presents a complex life cycle involving invertebrate triatomine hosts. Four main cellular forms can be identified during its life cycle: (i) the blood trypomastigote is the infective extracellular non-replicative form of the parasite, which is found in the bloodstream of the mammalian host, (ii) the metacyclic trypomastigote, also non-replicative, is present in the terminal portion of the digestive and urinary tracts of the vectors, (iii) the epimastigote is the replicative extracellular form of the parasite present in the triatomin insect vector, and (iv) the amastigote is the intracellular replicative form of the parasite in the vertebrate host (Rodrigues et al., 2014).

*Trypanosoma cruzi* is a paradigmatic case of predominantly clonal evolution (Tibayrenc et al., 1986) with “unequivocal evidence of genetic recombination” (Gaunt et al., 2003). Actually, the different known strains and clones of the parasite are classified into seven discrete typing units (DTUs), that are defined as a “sets of stocks that are genetically closer to each other than to any other stock and are identifiable by common molecular, genetic, biochemical, or immunological markers” (Tibayrenc, 1998). The DTUs ranged from *T. cruzi* I to *T. cruzi* VI, and lately, *T. cruzi* bat was also added (Zingales et al., 2012; Lima et al., 2015). Parasites from these DTUs are distributed differentially among insect and mammalian host species and therefore lives in different geographical areas (Zingales et al., 2012; Brenière et al., 2016). All *T. cruzi* parasites, regardless of the DTUs or where they belong, can cause CD. However, epidemiological studies suggest that *T. cruzi* I is associated with anthroponotic as well as sylvatic environments. On the other hand, *T. cruzi* II, V, and VI are mainly related to human environments and particularly chronic CD patients contrarily to *T. cruzi* III and IV which are found to be present in sylvatic habitats (Yeo et al., 2005). An association between DTUs and clinical outcomes is suspected (Messenger et al., 2015). These host and geographic specificities have been proposed to determine the probability of transmission and are related to the pathogenesis of CD. Parasites belonging to diverse DTUs have different biological properties, including growth rates in cultures, tropisms to tissue and organs, antigenicity, capacity to infect potential insect vectors, drug susceptibility, number of chromosomes, and DNA content (Macedo and Pena, 1998; Macedo et al., 2004). Characterization of the gene content and genome architecture of *T. cruzi*, as well as a whole-organism proteomic analysis of its four life cycle stages, has been reported (El-Sayed et al., 2005). Further, comparative genomics of different strains using different sequencing strategies and technologies provide data for the identification of genes associated with host tropism, pathogenicity, and modes of transmission (Berná et al., 2018; Callejas-Hernández et al., 2018; Reis-Cunha et al., 2018).

Whether all *T. cruzi* DTUs can be congenitally transmitted *in utero* is a matter of debate. There is presently no clear evidence that particular *T. cruzi* DTUs, as defined by the currently used molecular markers, would preferentially be transmitted congenitally [reviewed in Carlier and Truyens (2015), Truyens and Carlier (2017)]. We however, recently observed that congenital transmissions in Argentina and Mexico were associated with a maternal portage of “non-TcI” parasites (we did not detect congenital cases harboring TcI DTU) (Buekens et al., 2018). Concordantly, higher congenital transmission rates were reported in Brazilian regions where *T. cruzi* DTU V predominantly circulates as compared to a country where Tc II predominates (Luquetti et al., 2015).

## Parasite Tropism for Placenta

Tissue tropism to the placenta of different *T*. *cruzi* strains has been described previously, particularly in the murine model (Andrade, 1982). *T. cruzi* I (Colombiana strain) parasites present a high incidence of placental parasitism (98%) compared to *T. cruzi* II (Y strain) parasites that only infect 17% of the placentas. The same strains also present differences regarding the localization of the amastigotes in the placenta; only the Colombian strain could be observed consistently in the vascular part of the placenta. In a more recent work, chronic infected mice with two different *T. cruzi* strains, one obtained from a congenital CD patient [VD: Tc VI (Risso et al., 2004)] and the other one previously characterized as non-transmissible in mice [K98,Tc I (Solana et al., 2002)] showed, by means of DNA amplification and 18s ribosomal RNA expression studies, that VD displayed stronger placental tropism and lower parasitic loads in peripheral blood than the K98 strain (Juiz et al., 2017). On the other hand, female mice infected with the K98 strain present higher parasitemia than the ones infected with the RA strain (DTU VI). However, in RA infected females, the infected mice produced parasite-infected newborns, while K98 did not.

We also showed recently in human placental explants (HPE), as well as in a placenta-derived epithelial cell line (BeWo), that VD parasites present a higher infection capacity compared to the Y strain (Medina et al., 2018). These studies suggest that the parasite genotype plays a role in tissue tropism toward the placenta and might contribute to the probability of congenital transmission.

## Parasite Exovesicles as Virulence Factors

The persistence of *T. cruzi* parasites at a low level within specific tissues causes chronic pathogenic inflammation in some patients. How *T. cruzi* manages to persist and what factors released by the parasite influence its dynamics in tissue during chronic infection, remains poorly known (Teixeira et al., 2011). Nearly all cells can release extracellular vesicles, or exovesicles (EVs), which have been recognized as a mode of communication between cells. EVs may also participate in pathogenic processes (van Niel et al., 2006; Trocoli Torrecilhas et al., 2009; Cestari et al., 2012; de Pablos Torró et al., 2018). EVs are small membrane vesicles that are classified according to size, biogenesis, and composition, into exosomes and microvesicles (MVs). Exosomes, of 30 to 100 nm in size, are of endocytic origin, have a lipid bilayer, and are released into the extracellular compartment through the fusion of the multivesicular body (MVB) with the plasma membrane of the cell (Raposo and Stoorvogel, 2013). MVs are also referred to as ectosomes; are more heterogeneous in shape; and can vary between 100 and 300 nm in diameter. They are released as a result of the evagination toward the extracellular space from the plasma membrane. EVs differ not only in origin and size but also in lipid and protein composition. They have been identified in all biological fluids (Villagrasa et al., 2014), and their functions include intercellular communication, host-pathogen interactions, as well as the modulation of the immune response against infectious diseases or afflictions such as cancer (Schorey and Bhatnagar, 2008; Webber et al., 2015). We recently published reviews on the existence of EVs in protozoa and parasitic helminths and on the role of *T. cruzi* EVs (Marcilla et al., 2014; de Pablos Torró et al., 2018). Moreover, the induction of physiological modifications in cells by EVs derived from *T. cruzi* trypomastigotes has been recently demonstrated. These alterations involve the blocking of the cell cycle of the host cell, the permeabilization of the cells, and the disorganization of the cytoskeleton, among others (Retana Moreira et al., 2019).

EVs in *T. cruzi* were first described in 1970 (da Silveira et al., 1979) and found to be rich in glycoconjugates. Surface glycoconjugates are formed from glycoproteins (mucins) (de Lederkremer and Colli, 1995; Acosta-Serrano et al., 2001; Buscaglia et al., 2006; Mendonça-Previato et al., 2013), glycolipids (lipopeptidophosphoglycan-LPPG or glycoinosytolphospholipids-GIPL), and glycopeptides (NETNES) (Macrae et al., 2005). Electron microscopy and cryo-fracture have demonstrated that the vesicles come from the plasma membrane of the protozoan and the flagellar pocket. Recently, several surface proteins of the trans-sialidase, the cruzipain and the mucin-associated surface proteins (MASPs) families have been described in EVs, whose activity had previously been related to the modulation of the immune response by means of activating B cells and inducing a Th17 response or processes of adherence and cell invasion (Bermejo et al., 2011, 2013). Interestingly, MASPs are strongly expressed in the parasite and present a high variability between the different strains (Seco-Hidalgo et al., 2015). In recent works, we demonstrated that EVs, circulating in patients with CD, contained immature MASPs, with the presence of the C-proximal and N-terminal regions (peptide signal) (De Pablos et al., 2016; Díaz Lozano et al., 2017). MASPs are linked to the membrane through glycosylphosphatidylinositol (GPI) anchors. GPI participates in the inflammation processes that characterize CD (Almeida and Gazzinelli, 2001). The immature MASPs present in EVs (De Pablos et al., 2016; Díaz Lozano et al., 2017) are recognized by antibodies from CD patients. They help the parasite, in the presence of anti-*T. cruzi* antibodies, to evade the immune response by inhibiting the activity of C3 convertase and thereby impairing complement activation (Cestari et al., 2012). Similarly, the EVs isolated from the sera of CD patients are recognized both by the antibodies of the patients as well as by the serum of mice infected with the parasite. Therefore, it has been proposed that the immature MASPs in the outer side of the membrane of these EVs can form immunocomplexes with the IgGs of the patients (Díaz Lozano et al., 2017). In these studies, we demonstrate the involvement of EVs in the inhibition of the complement pathway, in which the terminal regions C- and N- of the immature MASPs participate. We also show how the antigens of the C- and N- immature regions of these MASPs inhibit the complement by antigen competition, in the presence of antibodies from CD patients. Besides, we identified differences in antigen recognition of the EVs by sera from the patients, depending on the pathology manifested by those affected by CD (De Pablos et al., 2016; Díaz Lozano et al., 2017). These results allowed us to consider *T. cruzi* EVs as markers of pathology, suggesting that they play a role in the pathogeny of CD. Their role in modulating the interactions between the parasite and the placenta and the congenital transmission is under investigation. However, preliminary results show that the parasite increases placenta-derived exosomes in HPE (Castillo et al., 2017a). The placenta is a rich source of exosomes which are related to fetal-placental-maternal communication, fetal allograft survival, and resistance to specific infections, among other functions (Ouyang et al., 2014; Schorey and Harding, 2016). Therefore, it is highly probable that placenta-derived exosomes play essential roles during *T. cruzi*-host interplay.

## Placental Responses to *T. cruzi*

The placenta is a temporary organ that separates the maternal and fetal compartments throughout pregnancy (Arora et al., 2017). The placenta is responsible for the metabolic exchange between mother and fetus, and fulfills endocrine and immune functions that ensure normal prenatal development of the fetus and pregnancy-related changes in the mother (Liempi et al., 2016; Arora et al., 2017). Mainly due to its regulatory role in the maternal/fetal immune response, this organ is able to protect the fetus against several pathogens (Delorme-Axford et al., 2014; Mor et al., 2017) (see below).

**FIGURE 1:**
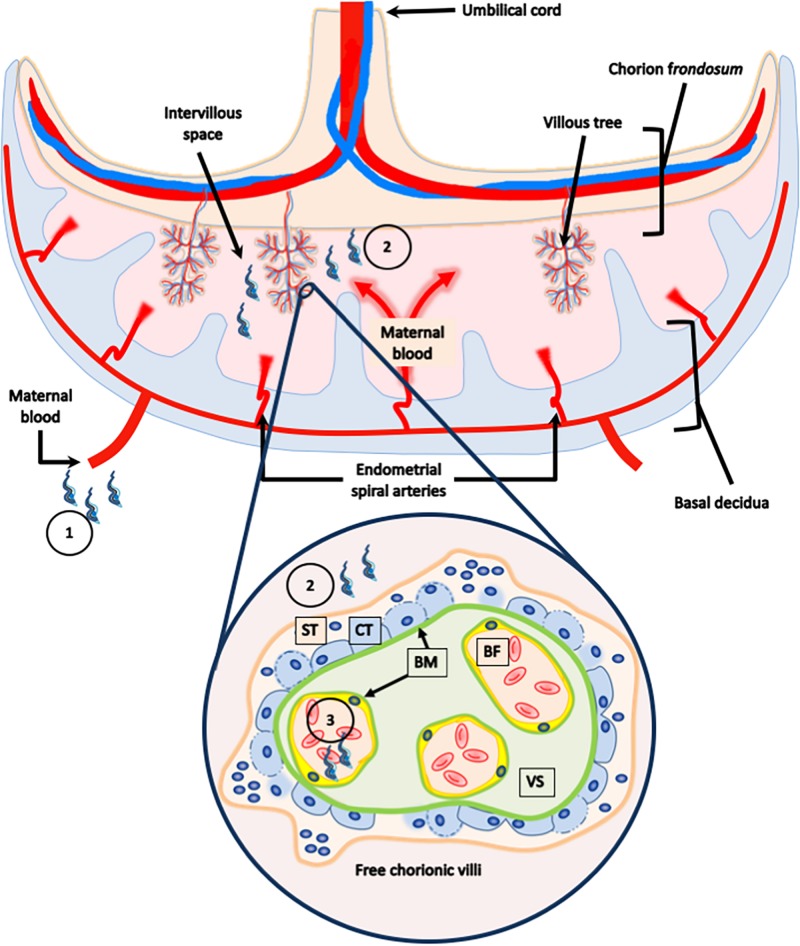
Human placental barrier. The human placenta is classified as a hemochorial chorioallantoic placenta. The placenta is composed of a fetal portion, developed from the chorion frondosum, and a maternal portion, or basal decidua, which originates from the endometrium. The functional units, were the placental barrier is located, are the free-floating chorionic villi formed by the trophoblast, and the villous stroma. Maternal blood surrounds and contacts the trophoblast in the placental intervillous space. The trophoblast is a bi-stratified covering epithelium composed of a superficial non-proliferative syncytiotrophoblast (ST), and a proliferate germinal layer, the cytotrophoblast (CT). The trophoblast is connected to and separated from the villous stroma (VS), the fetal connective tissue, and by a basal lamina. The parasite present in the maternal blood (1), that comes in contact with the trophoblast in the intervillous space (2), must cross the placental barrier in order to reach the fetal capillaries (3), and infect the fetus during transplacental transmission.

The human placenta is a highly invasive (hemochorial) chorioallantoic placenta. The functional units, where the placental barrier is located, are the free-floating chorionic villi formed by the trophoblast and the villous stroma. Maternal blood surrounds and contacts the trophoblast in the placental intervillous space (IVS). The trophoblast is a bi-stratified covering epithelium composed of a superficial non-proliferative syncytiotrophoblast (ST) and a proliferative germinal layer, the cytotrophoblast (CT). The trophoblast is connected to and separated from the villous stroma (VS), the fetal connective tissue by a basal lamina, a specialized structure of extracellular matrix (ECM) (Benirschke et al., 2012). Therefore, trophoblast, basal *laminae* and VS, the latter containing fetal capillaries, form the placental barrier that must be crossed by *T. cruzi* in order to infect the fetus during trans-placental transmission (Figure 1; Duaso et al., 2010; Carlier et al., 2012; Liempi et al., 2016). Of note, *T. cruzi* displays a high tropism for the decidual part of the placenta as compared to the heart, that is likely related to the local immune environment [see further and (Mjihdi et al., 2002)]. Also, in the placenta of mothers of congenitally infected infants, only a few amastigotes can be found in stromal cells such as macrophages, fibroblasts, and giant cells while they are not or scarcely found in the trophoblast, except in case of severe mortal cases of congenital infection (Carlier and Truyens, 2015).

Since the trophoblast is the first placental tissue in contact with the maternal blood, the ST is the first fetal cell layer exposed to the parasite. It has been demonstrated that a high concentration of *T. cruzi* trypomastigotes induces the destruction of the trophoblast in *ex vivo* infected HPE (Duaso et al., 2010). However, low parasite concentration induces cellular proliferation (Liempi et al., 2016; Droguett et al., 2017) and differentiation (Liempi et al., 2014, 2016) in the same placental tissue (see below). The basal *laminae*, located between trophoblast and the fetal connective tissue and around the fetal vessels are other structures that *T. cruzi* needs to overcome (Duaso et al., 2010). *T. cruzi* can bind to ECM molecules such as glycosaminoglycans, fibronectin, and laminin (Lima et al., 2002) using surface molecules such as gp85 (Maeda et al., 2014) and gp83 (Nde et al., 2006). The VS, the placental fetal connective tissue, is also an important obstacle for the parasite. The parasite presents several proteases, among which the cruzipain has been proposed to be responsible for collagen I destruction and disorganization in placentas from mothers with chronic CD or in *ex vivo* infected HPE (Scharfstein and Morrot, 1999; Castillo et al., 2012; Duaso et al., 2012; Maeda et al., 2014). Matrix metalloproteinases (MMPs) such as MMP-2 and MMP-9 are activated, and their expression levels are increased in the presence of the parasite. Interestingly, if these MMPs are inhibited, the parasite-induced damage in the placental tissue is prevented, and the presence of parasite DNA is decreased in *ex vivo* infected HPE (Castillo et al., 2012). Importantly, the ECM forms a complex tridimensional network with different types of elastic fibers and collagen molecules including collagen I fibers, glycoproteins, and proteoglycans (Duaso et al., 2010; Theocharis et al., 2019). Collagen I fibers are the main components of the ECM, and if these molecules are disorganized or destroyed, the 3D network of the ECM is also disrupted, a fact that could facilitate the parasite’s motility in the tissue, and its entrance into cells (Duaso et al., 2010). Moreover, *in vitro* studies have shown that during parasite-ECM interactions, the parasite changes its cytoskeletal conformation, the state of protease activation and adapts its metabolism (Mattos et al., 2019). In addition, parasite-induced ECM changes modulate the presence of cytokines and chemokines, allowing *T. cruzi* to manipulate and escape the innate and adaptive immune responses (Marino et al., 2003). Studies performed in CD patients with cardiac alteration have shown that *T*. *cruzi* derived-antigens promote a differential expression of MMP-2 and MMP-9. There is a positive correlation between MMP-2 and the immunomodulating cytokine IL-10, and a negative one with the pro-inflammatory cytokine IL-1β, whereas MMP-9 showed a negative correlation with IL-10. Therefore, it has been suggested that MMPs and cytokines produced in the myocardium in patients with CD are essential contributors to cardiac remodeling (Medeiros et al., 2017). It would be interesting to study the interactions between parasite-modulated MMPs and cytokines in the placenta.

Another placental response to the parasite is the trophoblast epithelial turnover. The epithelial turnover is part of the innate immune system. Pathogens adhere to the plasma membrane before invading host cells and, in case of a lining epithelium, they adhere to the cells of the superficial cell layer which is continuously removed. Therefore, the attached pathogens are removed with the superficial epithelial cells (Liempi et al., 2016).

The trophoblast turnover implies precise orchestration of different cellular processes: (i) cell proliferation of the CT, (ii) cellular differentiation (referring to the incorporation of CT cells into a non-replicative ST), and (iii) cell death by forming apoptotic bodies (knots) in the ST that counterbalance the proliferation of the CT cells. The apoptotic knots are released into the IVS where the maternal blood circulates (Benirschke et al., 2012; Mayhew, 2014; Liempi et al., 2016).

We have previously shown that *T. cruzi* induces the above-mentioned cellular processes related to the epithelial turnover in the trophoblast in both *ex vivo* infected HPE and *in vitro* infected BeWo cells (trophoblastic cell line) (Duaso et al., 2011; Liempi et al., 2014, 2016; Carrillo et al., 2016; Droguett et al., 2017). Thus, *T. cruzi* increases DNA synthesis as well as cellular proliferation markers such as PCNA (proliferating cell nuclear antigen) (Liempi et al., 2016). In BeWo cells, the parasite also increases the percentage of cells in the S and G_2_/M cell cycle phases and other commonly used proliferation markers (AgNORs and Ki67) (Droguett et al., 2017). It is noteworthy that PCNA modulates other cellular processes including DNA repair, cell cycle, survival, and gene expression (Wang, 2014), and that an increase of PCNA expression might occur in response to *T. cruzi*-induced damage. Regarding trophoblast differentiation, the parasite increases, in HPE and BeWo cells, the expression and or secretion of the main biochemical markers, including β-human chorionic gonadotropin (β-hCG) and syncytin (Liempi et al., 2014, 2016). Additionally, in BeWo cells *T. cruzi* induces cell fusion as demonstrated by the analysis of the re-distribution of desmoplakin (intercellular adhesion protein) and by a two-color fusion assay (Liempi et al., 2014). The parasite also activates the ERK1/2 MAPK pathway (Castillo et al., 2013b), one of the MAPK signaling pathways that mediate trophoblast differentiation (Forbes and Westwood, 2010). Finally, the parasite induces apoptotic cell death as part of trophoblast turnover (Liempi et al., 2016). Indeed, *T. cruzi* activates in HPE a caspase 3 like activity, followed by DNA fragmentation and pyknosis (Duaso et al., 2011). These data indicate that *T. cruzi* can boost all steps of the turnover of chorionic trophoblasts. Interestingly, a relation exists in trophoblasts between the cellular processes related to apoptosis and their differentiation (Gauster et al., 2009a, b). This is through caspase 8, a caspase operating upstream the pro-apoptotic caspase 3. Caspase 8 is transiently activated in CT cells just before their fusion into ST and is an essential protein in the process of formation of the ST barrier (Huppertz and Borges, 2008). We observed that *T. cruzi* infection increases the expression and activation of caspase 8 in BeWo cells as well as in HPE (Duaso et al., 2011; Carrillo et al., 2016). This fact might favor the integrity of the ST barrier as well as limit parasite entrance into ST (by favoring its turnover). Also, we showed that caspase 8 can slow down intracellular parasite multiplication (Carrillo et al., 2016), pinpointing caspase-8 activity as part of the trophoblast cell defense mechanisms against *T. cruzi* infection. Altogether, these results suggest that the trophoblastic response to *T. cruzi* may be a mechanism that reduces the risk of congenital transmission of the parasite. On the other hand, caspase 8 has recently been disclosed to display pro-inflammatory effects, by optimizing the cytokinic response to TLRs and by being able to activate the cytokine IL-1β (Lawlor et al., 2017). We speculate that such pro-inflammatory action causes placental lesions, favoring the trans-placental passage of *T. cruzi.*

As mentioned above, the placenta is an immune regulatory organ that modulates fetal as well as maternal immune responses (Mor et al., 2017). Pathogens are recognized by pattern recognition receptors (PRRs), including *Toll*-like receptors (TLRs). Activation of those PPRs results in the secretion of cytokines and chemokines aiming to fight infections (Janeway and Medzhitov, 2002). The human trophoblast expresses all ten of the known functional TLRs (Koga et al., 2009). *T. cruzi* is recognized by TLR-2 and TLR-4 (present at the cell surface) as well as the endosomal TLR-7 and TLR-9 (Tarleton, 2007; Gravina et al., 2013; Castillo et al., 2017b). We have shown, in HPE, that *T. cruzi* infection induces protein expression and activation of TLR-2, but not of TLR-4 and TLR-9 (Castillo et al., 2017b). *T. cruzi* induces the secretion of IL-1β, IL-6, IL-8, IL-10, and TNF-α in HPE (Castillo et al., 2017b). Of note is that secretion of IL-1β, IL-6, and TNF-α have been associated, within the trophoblast, with cellular proliferation, and differentiation (Haider and Knöfler, 2009; Hamilton et al., 2012). Interestingly, the inhibition of TLR-2 impairs trophoblast turnover and increases parasite infection (Castillo et al., 2017c). However, to date we do not know if cells other than trophoblasts, such as macrophages in the VS, are also involved in TLR activation. However, our results allow us to propose TLR-2 initiated cytokine profile as a local placental defense mechanism.

## Transcriptomic Studies in Placentas in Response to *T. cruzi* Infection

The global placental transcriptomic response to *T. cruzi* infection has also been studied. This approach allows us to study the gene expression profiles during infection, allowing the identification of new genes and/or pathways implicated in the establishment of the infection and pathogenesis. The RNA-seq study has been carried out in placentas obtained from term deliveries in *T. cruzi*-infected and non-infected women. Forty-two differentially expressed genes (DEGs) were identified, and gene-set association analysis was performed to detect pathways linked to parasite infection. It showed, not surprisingly, that the inflammatory and the immune responses were upregulated in infected placentas while the anti-inflammatory cytokine IL-38 (formerly IL-1 family member 10-IL1F10) (Garraud et al., 2018) was under-represented (Juiz et al., 2018). Placental inflammation is in line with other reports showing that: (i) newborns of *T. cruzi*-infected mothers are prone to produce higher levels of pro-inflammatory cytokines in comparison to those born to non-infected mothers (Vekemans et al., 2000), (ii) *T. cruzi* infection of pregnant women affects the developing immune system of fetuses independently of congenital infection (Dauby et al., 2009), which might be related to placental inflammation (Jennewein et al., 2017), and (iii) the parasite induces, as mentioned above, in *ex vivo* infected HPE, a highly significant increase of pro-inflammatory and immunomodulatory cytokines (Castillo et al., 2017b,c).

Analysis of DEGs also suggests that the placental inflammation might be counter-regulated in infected mothers. For instance, placentas from infected women displayed lower expression of genes related to exocytosis pathways and neutrophil degranulation, probably limiting damages associated with inflammation. Moreover, the expression of the immunomodulatory HLA-G was increased (Juiz et al., 2018). HLA-G is responsible for the generation of immunological tolerance during gestation by inducing regulatory T cells and tolerogenic dendritic cells (Carosella, 2011; Ferreira et al., 2017) and might increase susceptibility to infections. Hence, in malaria, increased levels of HLA-G in infected mothers are associated with an increased risk to acquire malaria during infancy (Sadissou et al., 2014). S100A14 is another interesting parasite-modulated gene, that is downregulated in placentas from women with CD. This gene codes for a member of the S100 protein family that regulates cell cycle progression, cellular differentiation, and triggers an inflammatory response by engaging the receptor for advanced glycation end products (RAGE) (Jin et al., 2011). Interestingly, this same protein, S100A14, is reported to increase the expression of the collagenase MMP2 (Chen et al., 2012; Qian et al., 2016), which as explained above regulates parasite infection. This might be in line with the observation that mutations on the MMP2 gene favor congenital *T. cruzi* transmission (see hereunder). On the other hand, PRG2 a gene that encodes the precursor form of the eosinophil major basic protein (proMBP) is an upregulated DEG in *T. cruzi*-infected placentas. MBP can participate in the extracellular killing of parasites in the absence of antibodies (Nakhle et al., 2018). Another important function of MBP in the placenta is the inhibition of the pregnancy-associated plasma protein A (PAPPA) (Weyer and Glerup, 2011), whose low levels are associated with intrauterine growth restriction (Albu et al., 2014). Therefore, overexpression of the PAPPA inhibitor in the placenta of women with CD might also play a role in the congenital transmission of *T. cruzi*.

Other, DEGs observed in placentas from seropositive women and related to pregnancy were the genes CGB5 and KISS1, which encode for hCG and kisspeptin, respectively. Both genes were downregulated, and low serum levels of both proteins have been proposed as markers of miscarriage (Jayasena et al., 2014). Low KISS1 expression is associated with recurrent pregnancy loss as well as preeclampsia and intrauterine growth restriction (Armstrong et al., 2009; Park et al., 2012). In contrast, the DEG TAC3 gene [encoding neurokinin B (NKB)] was upregulated in placentas from seropositive women. NKB overexpression in the trophoblast is associated with a decreased blood flow to the placenta and increased vasoconstriction in the endometrium and therefore related to preeclampsia (Familari et al., 2017). Interestingly, NKB and kisspeptin have a role in hCG placental expression in response to estradiol (Oride et al., 2015) and hCG, as described above, is induced by *T. cruzi* in HPE and BeWo cells (Liempi et al., 2014).

A microarray-based transcriptomics study carried out in *ex vivo* infected HPE (Castillo et al., 2018), corroborates most data obtained by RNA-seq in whole placentas (Juiz et al., 2017, 2018) as well as the *T. cruzi-*invasion mechanisms in HPE. Genes that are involved with ECM remodeling are upregulated, in concordance with our previous findings that demonstrate the parasite-induced expression and activation of MMP-2 and MMP-9 (Castillo et al., 2012). The changes in gene expression regarding signal transduction pathways were also confirmed (Castillo et al., 2013b, 2017b). Genes involved in innate immunity were overexpressed, including CD46 and C1q that regulate or form part of the complement system. Upregulation of C1q might have an impact since it binds the *T. cruzi* calreticulin, thereby enhancing the infection (Castillo et al., 2013a). On the other hand, TLR-7 and TLR-8 are mainly increased while TLR-2, whose inhibition favors infection and tissue damage (Castillo et al., 2017c), appeared not to be upregulated in the microarray analysis. Another contradictory observation was that a high *T. cruzi* concentration decreased IL-6 expression more than 60-fold as compared to control non-infected explants, whereas no changes were observed when HPE were infected with a low parasite concentration. On the other hand, mRNA expression analysis showed a higher level of transcription, suggesting that regulation of IL-6 could occur at post-transcriptional stages (Castillo et al., 2017c).

Furthermore, similar to the study in whole placentas, the majority of overexpressed genes were related to fetal development and pregnancy-related processes, particularly *GH2*, *CSH1*, and *CSH2* genes that encode the pregnancy-specific beta-1-glycoproteins growth hormone 2, chorionic somatomammotropin hormone 1 and 2, respectively.

Another microarray-based study was done in placental tissues from C57Bl/6J mice chronically infected with two distinct *T. cruzi* strains (VD and K98, see above) (Juiz et al., 2017) A total of 247 DEGs were identified between infected and non-infected mice: 140 and 107 genes were up- and genes downregulated, respectively, compared to non-infected mice. Placentas infected with the VD parasites showed a higher number of DEGs compared to the K98 infected ones (211 vs. 89 DEGs). There were DEGs common to both infected groups and specific ones for each infected group; while 69% (59/89) of genes were downregulated by K98 infection, VD infection produced this response only in 39% (83/211) of genes. Analysis of DEG networks by GeneMANIA showed that the “Secretory Granule” pathway was downregulated in both infected groups, whereas “Response to Interferon-gamma” as well as “Innate Immune Response” pathways were upregulated only in placental tissues infected with VD. This is interesting since, as said above, the K98 strain displays poor placental tropism and no congenital transmissibility, contrary to the VD strain. It suggests the role of placental inflammation in the process of congenital transmission. Another analysis that detects small changes in predetermined gene sets (Gene-Set Enrichment Analysis algorithm) showed downregulation of genes involved in transcription, macromolecular transport, and metabolism in infected placentas and upregulation of genes regulating signal transduction pathways. Genes regulating apoptotic cell death were downregulated in the murine placentas infected with both parasite strain.

These transcriptomic analyses identify a gene expression profile in the placentas from *T. cruzi*- seropositive mothers that are different from those from seronegative women. It globally shows that affected genes are involved in cell adhesion, cellular proliferation and differentiation, apoptosis, vesicle transport processes, ECM organization, lipid and protein metabolism, and the inflammatory/immune responses.

## Family Clustering of Congenital Transmission of *T. cruzi* Infection and SNPs

It is still unknown why some infected mothers transmit the infection in successive gestations to their babies while others do not, leading to family clustering of congenital transmission. A case-control study of single-nucleotide polymorphisms (SNPs) located within human loci encoding different proteins expressed in placental tissues was performed using genomic DNA from clinical samples of 116 non-infected children born to seropositive women and children with congenital *T. cruzi* infection. Logistic regression analysis showed that susceptibility of congenital infection was associated with SNPs in sites rs243866, rs17859821, and rs2285053from MMP2 gene and rs11244787 and rs1871054 sites from the ADAM 12 locus. In case of MMP2 rs243866 and rs17859821 positions, one or both copies of the mutant allele (Adenine) increased the likelihood of congenital infection, whereas, for rs2285053, both T alleles are needed for susceptibility to infection. Mutation in rs2285053 interrupts a CCACC box promoter site (Sp1-type) causing a weaker activity of the promotor. The rs243866 position is located upstream of a half-palindromic potential estrogen receptor binding site (Harendza et al., 2003). Both rs243866 and rs2285053 mutant alleles reduce the transcription activity of MMP2, which in turn modulate the ECM-remodeling and immune responses, and thus the susceptibility to infection. ADAM12 belongs to the ADAM protein family; it is a membrane-bound MMP-protease with a role in cell-cell and cell-matrix interactions that are associated with muscle development, fertilization, neurogenesis inflammatory, and immune responses. In preeclampsia (Grill et al., 2009) and ectopic pregnancy (Rausch and Barnhart, 2012), among other disorders related to pregnancy, ADAM 12 plasmatic concentrations are altered. Also, ADAM 12 promotes the transforming growth factor β (TGF-β) signaling activation leading to transcriptional activation. Importantly, TGF-β activation increases host cell susceptibility to *T. cruzi* infection (Ming et al., 1995; Hall and Pereira, 2000).

Various other SNPs have been related to the susceptibility to *T. cruzi* infection and progression of CD. Particularly, genes encoding cytokines/chemokines involved in inflammatory and immune response have been proposed as biomarkers (Calzada et al., 2009; Torres et al., 2010; Pissetti et al., 2011; Flórez et al., 2012; Nogueira et al., 2015; Furini et al., 2016; Ferreira et al., 2018). Thus, TGF-β1 polymorphism, mainly CT and TT genotypes at position – 509 of the TGFB1 gene have been associated with the susceptibility of acquiring CD in a Brazilian population (Ferreira et al., 2018). The same genotype, in addition to the genotypes −988 C/A; −800 G/A; −10 T/C; and 263 C/T has been studied in the Colombian and Peruvian populations. In this study, the genotype 10 C/C was increased in the group of CD patients of both populations and has been proposed to be involved in differential susceptibility to *T. cruzi* infection (Torres et al., 2010). Another important pro-inflammatory cytokine involved in CD is TNF-α. The TNF- α gene is located in the MHC locus, and its polymorphism is associated with many infectious diseases, including parasitic ones. TNF-α is increased in the hearts of patients with chronic chagasic cardiomyopathy and is associated with tissue damage. An association was observed between the absence of the TNF-238A allele and negative serology for CD. Seropositive individuals carrying the TNF-238A allele produced significantly higher TNF-alpha levels than healthy ones and therefore the TNF-α polymorphism at position −238 has also been associated to the susceptibility to *T. cruzi* infection and CD progression (Pissetti et al., 2011). Other SNPs in genes encoding for cytokines and molecules involved in the immune response has been related to CD, including interferon (IFN)-γ (-874 T/A) (Torres et al., 2010) and IL-18 (rs2043055 polymorphism) (Flórez et al., 2012; Nogueira et al., 2015), RANTES, and chemokine receptors CCR2 and CCR5 (Flórez et al., 2012). It would thus be worthwhile to also investigate such polymorphisms in the context of congenital transmission of *T. cruzi.*

## Maternal *T. cruzi* Systemic Immune Response, Gestation, and Congenital Infection

The immune system during pregnancy is characterized by a subtle balance between immune tolerance and activation. Regulatory T cells are increased during pregnancy in order to avoid fetal rejection. Indeed, the maternal immune cells recognize paternal antigens expressed by the trophoblast and other fetal cells crossing the placenta to the maternal circulation. The mechanisms leading to this regulatory environment have been largely described elsewhere (Aluvihare et al., 2004; Racicot et al., 2014). Maternal immune tolerance is fundamental to a successful pregnancy. It does, however, not mean that the pregnant women are unable to mount immune responses, and trophoblasts possess multiple ways to escape immune attack (Straszewski-Chavez et al., 2004). Besides, immune responses during gestation are, except for the periods of implantation, placentation, and parturition, physiologically oriented toward a Th2-type. It likely aims to protect the uteroplacental unit and the fetus against harmful inflammation (Mor et al., 2017). Whereas the regulatory and Th2-biased environments are particularly pronounced at the level of the UPU, it may also impact peripheral, and systemic immune responses (Reinhard et al., 1998).

*Trypanosoma cruzi* infection induces a complex immune response. The control of *T. cruzi* infection relies on diverse effector mechanisms needed to fight both extracellular trypomastigotes and intracellular amastigotes. Therefore, IFN-γ, cytotoxic cells, and antibodies (Abs) all play a role in the control of the infection [reviewed in Truyens and Carlier (2017)]. Antibodies potentially participate in extracellular parasite elimination mainly by inducing complement-dependent and independent lysis of parasites and phagocytosis of opsonized parasites. IFN-γ, initially produced by NK cells, then by both CD4 + Th1 and CD8 + T cells, is a significant player in the immune responses against intracellular pathogens (Kak et al., 2018). It activates among other the microbicidal properties of phagocytes, mainly macrophages, allowing them to limit and/or kill the intracellular parasites. Infected cells can be killed by cytotoxic CD8 + T cells while the cytotoxic action of NK cell on *T. cruzi*-infected cells is likely not significant (Truyens and Carlier, 2017). The cellular response to *T. cruzi* is thus mainly Th1-oriented. Our studies in mice show that this inflammatory immune response has serious harmful effects on gestation when the host is in the acute phase of infection. Indeed, acute infection impedes reproduction, related to either infertility induced by the infection (inhibition of cell division of the embryo before implantation) or fetal loss occurring along gestation, due to massive placental invasion by parasites. Chronic maternal infection does not affect reproductive capacity but induces reversible fetal growth retardation (Carlier et al., 1987). Such deleterious effects did not rely on congenital infection but were associated with increased production of TNF-α (Rivera et al., 1995; Mjihdi et al., 2002). In humans, most women of gestational age have a chronic infection (see above). We have similarly observed that *T. cruzi*-infected women give birth to newborns with low birth weight, in the absence of congenital infection more frequently (Torrico et al., 2004).

Besides, in the absence of congenital transmission, several data indicate bidirectional and opposite interactions between the immune response to *T. cruzi* and the immune environment of gestation. On one side, pregnancy dampens and modifies the immune response to *T. cruzi*. Indeed, we found that blood cells from chronically infected pregnant women released, on average, two to threefold less IFN-γ and IL-2 in response to non-specific lymphocyte stimulants (PHA and LPS) than non-pregnant women (Hermann et al., 2004). They also produced, on average, sevenfold less IL-10. Accordingly, lower levels of IL-10 and IFN-γ were detected in the serum of pregnant infected women than in non-pregnant infected ones (Cardoni et al., 2004). This shows that pregnancy reduces the global capacity of their lymphocytes to be activated (though it is not completely inhibited), likely as a result of the relative immunosuppression associated with gestation. Also, the cytokine profile moved toward a more inflammatory environment in infected pregnant women, since the capacity to produce the inflammatory cytokine IFN-γ was less inhibited during pregnancy than the capacity to produce IL-10 (an anti-inflammatory cytokine), being in line with the inflammatory transcriptome detected in the placenta of *T. cruzi*-infected women [see above (Juiz et al., 2018)]. On the other hand, Egui et al. recently observed in a murine experimental *T. cruzi* infection model that chronic infection reduced the expression of gestation-induced inhibitory receptors on T cells (CTLA4 on CD4 + and CD8 + T cells and CD160 on CD8 + T cells), suggesting that *T. cruzi* chronic infection counteracts the immunosuppression associated with gestation but does not impact the gestation outcome (Egui et al., 2017).

Our previous studies in pregnant chronically infected women showed that gestation also reduced the specific IFN-γ response to parasite antigens as compared to the response of non-pregnant infected ones (Hermann et al., 2004). This was the case for women that transmitted (T mothers) as well as those that did not transmit (NT mothers) the parasite to their fetus. Strikingly, this specific IFN-γ response was markedly lower in T than NT mothers and was associated with less activated monocytes (Hermann et al., 2004). On the other hand, we and others have reported that the circulating parasite load is slightly increased during the second and third trimester of pregnancy (Brutus et al., 2010; Siriano et al., 2011). Moreover, T mothers displayed higher parasitemia than NT ones (Hermann et al., 2004; Kaplinski et al., 2015). IFN-γ is well known as a key factor in the control of *T. cruzi* infection (Truyens and Carlier, 2017). These data strongly suggest that the increase of parasite burden in T mothers results from a reduced control of the infection and points to a central role of parasite burden as a risk factor of congenital transmission. In line with this, several studies underline that congenital transmission occurs mostly in mothers displaying blood parasites amounts that are detectable by PCR (i.e., PCRs are generally negative in NT mothers). The microbial burden is also a key factor of congenital transmission of other pathogens (Lilleri and Gerna, 2017).

The increase of parasite burden during gestation likely results from the relative immunosuppression associated with gestation, while the increased parasite load in T mothers probably relates to their more depressed IFN-γ response. The question is, thus, why T mothers display such feature? Several factors may be considered, concerning the parasite, the host, or epidemiological parameters. Living in endemic countries where vectorial transmission occurs may lead to frequent reinfections. Multiple infections, though leading to transiently increased parasite levels, result in lower parasitemia in the long term, the hypothesis being that repeated antigen exposure reinforces the Th1 response that controls the parasite (Machado et al., 2001; Bustamante et al., 2007; Kaplinski et al., 2015). In line with this, it was observed that higher vector exposure of women living in endemic countries decreases the risk of congenital transmission (Sánchez Negrette et al., 2005; Kaplinski et al., 2015). Congenital transmission rates are also lower in non-endemic countries (2.7%), free of vectorial reinfections, than in endemic countries [2.7% vs. 5%, respectively (Howard et al., 2014)]. Parasite load may also vary according to the parasite genotype/strain (Moreira et al., 2013).

Our data also suggest that host factors modulate the risk of congenital transmission of *T. cruzi*. Genetic and epigenetic factors may influence the efficacy of immune responses against pathogens and consequently modulate the microbial load (Möller et al., 2018). For instance, polymorphism of genes encoding proteins of the axis IL-12-IFN-γ and genetic variations affecting the modulatory action of long non-coding RNAs or micro-RNAs are reported to impact diseases (Vannberg et al., 2011; Duval et al., 2017; Gao and Wei, 2017; Ellwanger et al., 2018). We compared the ability of T and NT women to produce IFN-γ to *T. cruzi* and mitogens several months after they had given birth, i.e., in the absence of the immunosuppression of pregnancy (Hermann et al., 2004). We found that T women still displayed a more depressed ability to produce this cytokine than NT women, suggesting that inherent host factors involved might be genetic. Likewise, we have recently reported that several genes related to the immune system were differentially expressed in the placenta of T and NT mothers as well as an association between congenital *T. cruzi* transmission and the presence of polymorphism among some gene expressed in the placenta (see above) (Juiz et al., 2016, 2018). Of note, differential immune responses associated with the congenital transmission of other pathogens have also been recently reported (Lilleri and Gerna, 2017; Wujcicka et al., 2018). Besides, epigenetic modulation may also have occurred in some women in relation either with the *T. cruzi* strains present in the host or to their history of infections with other pathogens (Gomez et al., 2013; Smith and Denning, 2014).

Finally, we and others noticed that T mothers were younger and had a lower mean number of previous pregnancies than the NT mothers (Hermann et al., 2004; Kaplinski et al., 2015), which might be related to the immuno-enhancing effect of multiparity on the maternal immune system (Skowron-Cendrzak et al., 1999).

## Neonatal Immune Response to *T. cruzi*

Immune responses in early life are physiologically different from adult responses. Similarly, to what occurs during pregnancy, the neonatal immune system is characterized by a certain degree of immunodeficiency and weak Th1 but excessive Th2 responses, contributing to high susceptibility to pathogens and the development of suboptimal immune responses to vaccines administered in early life. However, the mechanisms leading to immunodeficiency and Th2 bias are completely different from those activated during pregnancy and have been reviewed elsewhere (Levy, 2007; PrabhuDas et al., 2011; Elahi et al., 2013). Mechanisms responsible for the regulatory environment comprises, among others, impaired signaling pathways downstream of *Toll-like* receptors 3 and 4, the presence of higher circulating levels of adenosine, the presence of higher numbers of regulatory T cells, and the presence of particular inhibitory “erythroid cells.” The Th2 bias results from the hypermethylation of the *IFNG* gene, while the Th2 locus is hypomethylated. Also, the proliferation of Th1 T cells in early life is strongly hindered due to the expression of a particular receptor for IL-4 produced by Th2 T cells. Engagement of this receptor induces apoptosis of Th1 cells.

Our studies in humans, performed in infants from chagasic mothers, point out that *T. cruzi* infection triggers neonatal type 1 immune responses, overcoming the physiological immune deficiency associated with early life (Hermann et al., 2002). Indeed, CD8 + T cells, and CD4 + T cells to a lesser extent, were activated in *T. cruzi* congenitally infected new-borns to an adult-like level and produced IFN-γ. Besides, their NK cells display phenotypic and functional alterations suggestive of a previous *in utero* activation when parasites were transmitted from the mother (Hermann et al., 2006). It also pinpoints the imprinting of the maternal *T. cruzi* infection on the neonatal immune system, revealing an immuno-stimulatory/adjuvant property of the parasite, as both congenitally infected and uninfected infants from chagasic mothers responded more strongly to vaccines directly administered during the first 6 months of life, like those against BCG, hepatitis B, tetanus, and diphtheria (Dauby et al., 2009). In trying to decipher the mechanisms allowing the parasite to induce type 1 immune responses in early life, we showed that the parasite could strongly activate neonatal NK cells to produce IFN-γ, known to drive Th1 type responses rapidly. NK cell activation by *T. cruzi* is indirect and depends on cross-talk with monocytes (and not with dendritic cells), on IL-12 synthesis and engagement of TLR2, 4, 7, and 9 (Guilmot et al., 2013). The parasite also very efficiently licensed neonatal dendritic cells to activate CD4 + and CD8 + T cells. Interestingly, DC and monocyte activation by *T. cruzi* is reinforced in the presence of maternal IgG isolated from cord blood samples from neonates born to NT infected mothers, i.e., carrying *T. cruzi*-specific IgG (Rodriguez et al., 2012). These observations allow us to make the hypothesis that monocytes, likely activated in fetuses from infected mothers combined with the maternally transmitted *T. cruzi* – specific antibodies might allow the fetus to fight parasites and maybe in some cases to eliminate them if only a few parasites were transmitted.

## Final Remarks

Some relevant questions regarding the role of parasite diversity, host genetic response, and host immune responses deserve further investigations. The study of gene expression profiles during infection constitutes a powerful tool to analyze global responses of several kinds of cells and tissues, allowing the identification of new genes and/or pathways implicated in the establishment of the infection and pathogenesis as well as possible local tissue responses. Other aspects such as the role of maternal microbiomes in the likelihood of vertical transmission of *T. cruzi* have not been investigated yet.

## Author Contributions

All authors listed have made a substantial, direct and intellectual contribution to the work, and approved it for publication.

## Conflict of Interest Statement

The authors declare that the research was conducted in the absence of any commercial or financial relationships that could be construed as a potential conflict of interest.
